# Pediatric Lyme Disease in Northern Italy: An 18-Year Single-Center Case Series

**DOI:** 10.3390/microorganisms12030455

**Published:** 2024-02-23

**Authors:** Federica Forlanini, Raffaella Di Tonno, Roberta Caiazzo, Daniela David, Maria Sole Valentino, Sara Giordana Rimoldi, Gian Vincenzo Zuccotti, Giusto Trevisan, Francesca Wanda Basile, Vania Giacomet

**Affiliations:** 1 Department of Pediatrics, V. Buzzi Hospital, Università degli Studi di Milano, Via L. Castelvetro 32, 20133 Milan, Italy; federica.forlanini@unimi.it (F.F.); gianvincenzo.zuccotti@unimi.it (G.V.Z.); 2 Pediatric Infectious Disease Unit, Università degli Studi di Milano, L. Sacco Hospital, Via G.B. Grassi 74, 20157 Milan, Italy; ditonno.raffaella@asst-fbf-sacco.it (R.D.T.); caiazzo.roberta@asst-fbf-sacco.it (R.C.); david.daniela@asst-fbf-sacco.it (D.D.); valentino.maria@asst-fbf-sacco.it (M.S.V.);; 3 Microbiology, Virology and Bioemergency Unit, ASST Fatebenefratelli Sacco, 20157 Milan, Italy; rimoldi.sara@asst-fbf-sacco.it; 4 Department of Medical Sciences, University of Trieste, 34100 Trieste, Italy; trevisan@units.it

**Keywords:** borreliosis, pediatric, surveillance, Lyme, infection, erythema migrans, child

## Abstract

Tracing the profile of pediatric Lyme borreliosis (LB) in Europe is difficult due to the interregional variation in its incidence and lack in notifications. Moreover, the identification of LB can be challenging. This study is an 18-year case series of 130 children and adolescents aged under 18 years referred to the Pediatric Infectious Diseases Unit at L. Sacco Hospital, Milan, with suspicion of LB, between January 2005 and July 2023. The routine serological workup consisted of a two-step process: an initial screening test followed by Western blot (WB). Forty-four (34%) patients were diagnosed with LB. The median age was six years, and 45% were females. Of the children with erythema migrans (EM), 33 (57%) were confirmed as having true EM, and, of these, 4 (12%) were atypical. Ten (23%) patients had early disseminated/late diseases, including facial nerve palsy (*n* = 3), early neuroborreliosis (*n* = 1), arthritis (*n* = 3), relapsing fever (*n* = 2), and borrelial lymphocytoma (*n* = 1). No asymptomatic infections were documented. Over seventy percent of confirmed LB cases (*n* = 31/44) recalled a history of tick bites; in this latter group, 19 (61%) were from the area of the Po River valley in Lombardy. Almost half of the children evaluated for LB complained of non-specific symptoms (fatigue, musculoskeletal pain, skin lesions/rash, and persistent headache), but these symptoms were observed in only two patients with confirmed LB. Most LB cases in our study were associated with EM; two-tier testing specificity was high, but we found frequent non-adherence to international recommendations with regard to the timing of serology, application of the two-step algorithm, and antibiotic over-prescription. Most children were initially assessed for a tick bite or a skin lesion suggestive of EM by a family pediatrician, highlighting the importance of improving awareness and knowledge around LB management at the primary healthcare level. Finally, the strengthening of LB surveillance at the national and European levels is necessary.

## 1. Introduction

Lyme borreliosis (LB) is a multisystem zoonotic disease caused by a distinct group of spirochetes belonging to the family Borreliaceae, often referred to as “*B. burgdoferi sensu lato*” [1]. The pathogen is transmitted to humans by ticks of the Ixodes genus. The commonest species in Europe is *Ixodes ricinus*, with the predominant borrelial genospecies carried being *B. afzelii* and *B. garini* [2,3]. Children are commonly infected in the spring and summer months during outdoor activities and when ticks are most active [4]. 

The real incidence of LB is unknown, and has likely been underestimated until recent years, when there has been a remarkable increase in serologically confirmed diagnoses and global awareness [5]. In Europe, there is an average of 65,400 LB cases per year, with significant discrepancies between countries [6,7,8]. In Western Europe, the rates of pediatric LB range between 9 and 52 cases per 100,000 children per year [9]. However, tracing the profile of pediatric LB disease in Europe is difficult, not only due to the expected interregional variation in its incidence and seroprevalence, but also because many countries do not actively contribute to the European surveillance of LB, and data are often not disaggregated by age [6,10]. Therefore, there is a lack of information and inhomogeneity about pediatric case definitions, notification criteria, and reporting methods. 

Italy is thought to have one of the lowest LB rates among the European countries [11]. In a recent study that included children, the annual incidence in the Northern Italian region of Lombardy in the years 2000–2015 exhibited broad temporal and geographical variation, with the lowest incidence of 0.3 cases per one million residents occurring in 2005, and the highest of 2.6 cases per one million residents in 2014 [12]. Remarkably, the provincial incidence rates demonstrated even more pronounced variability, with the lowest incidence of new cases per year at 0.3 occurring in Lodi and the highest peaking at 7.6 in the Sondrio provinces [12]. One recent spatiotemporal analysis of LB in the same region confirmed the widespread presence of Ixodes populations, suggesting that the incidence of LB in Northern Italy was higher than previously thought [13].

The diagnosis of LB should be based on a suggestive history of tick exposure, consistent symptoms, and serology. However, the identification of LB can often be challenging due to non-specific symptoms and a lack of history of tick exposure, especially in children [14]. Typically, LB can be divided into three distinct time- and symptom-based phases: early localized (corresponding to erythema migrans, EM), early disseminated, and late disease. Classic EM is pathognomonic of early LB; its diagnosis can rely on this clinical finding alone, and specific antimicrobial treatment can be started immediately [15]. In the early disseminated stage, which occurs 3 to 12 weeks after infection, symptoms include malaise, fever, neurological issues, muscle pain, and cardiac symptoms. Joint involvement is common, and some patients may experience CNS issues like encephalopathy and Bell’s palsy. Late Lyme disease, occurring months or years later, involves neurological and rheumatological symptoms [16].

Serology is not indicated by the presence of EM, as sensitivity can be low in the early stages of LB but is required in the suspicion of all other forms of disease [17,18]. International guidelines recommend a two-step diagnostic approach also known as two-tier testing: an enzyme-linked immunoassay (EIA) or immunofluorescence assay (IFA) as the first step and, in cases of a positive result, a confirmatory immunoblot. For a positive immunoblot, the presence of 2 out of 3 IgM bands, or 5 out of 10 IgG bands, is required [19]. The diagnostic utility of IgM is limited by low sensitivity in the early stages of the disease (i.e., when EM is present), and low specificity in chronic illness, due to a high number of false positives. For these reasons, their use is recommended only in the first 4 weeks after disease onset [20,21,22]. Furthermore, an analysis of cerebrospinal fluid (CSF) with an assessment of intrathecal antibody production (CSF/serum antibody index) should be performed in all cases of suspected neuroborreliosis [23].

Given the complexity of the diagnosis of LB and test interpretation, especially in late disease, clinicians’ awareness of the risk of infection and familiarity with testing approaches are fundamental, especially in non-endemic areas where the pretest probability of infection is low. 

The aim of this study is to describe the clinical and laboratory characteristics of an 18-year case series of pediatric patients referred to the Pediatric Infectious Diseases Unit of Luigi Sacco University Hospital, Milan, with suspicion of LB.

## 2. Materials and Methods

### 2.1. Study Design, Patients, and Setting

This is a retrospective analysis of medical records of all children and adolescents aged below 18 years referred to the Pediatric Infectious Diseases Unit at Luigi Sacco Hospital, part of Milan University, with suspicion of LB, between January 2005 and July 2023. Milan, located in the Lombardy region, is the largest city in northern Italy, and the center of a densely populated urban area between the Alpine arc and the Po River. Luigi Sacco Hospital is the regional Referral Center for Infectious Diseases in Lombardy, hosting since 2015 a multidisciplinary team for the diagnosis, treatment, and overall care of patients with LB. The team includes pediatricians, infectious diseases specialists, neurologists, dermatologists, and a clinical microbiologist. 

Children were referred to our facility by their general pediatrician or by an emergency department physician from Lombardy and neighboring regions. We included patients who were referred with a provisional diagnosis of LB or suspected LB based on clinical or laboratory grounds, and patients who underwent LB testing as part of their hospital workup.

The following variables were collected from each patient’s medical record: history and geography of the tick bite, presenting symptoms, serological testing, treatment, and outcomes. De-identified data were extracted manually from patients’ medical files by pediatricians collaborating with the study and anonymously input on a password-protected digital worksheet.

### 2.2. Definitions

EM was diagnosed as per the Centers for Disease Control and Prevention (CDC) definition: classic EM was defined as a flat, non-itchy, ring-shaped skin lesion usually located at the site of the bite, which could develop in the range of 3 to 30 days after the bite (average of 7 days), gradually expanding over time and sometimes resulting in a target-like appearance [24]. We defined as “atypical” any EM not fully meeting the CDC definition. Fever was defined as the presence or history of body temperature >37.5 °C for at least 3 days. Recurring febrile episodes that lasted for approximately 3 days and were separated by 7-day afebrile periods were defined as relapsing fever [25]. Patients with a confirmed history of a tick bite but with no observable symptoms or signs were defined as “asymptomatic tick bite”. Patients with positive two-tier testing and without clinical signs and symptoms were defined as “asymptomatic infection”. We then classified Lyme neuroborreliosis into three levels of certainty, in accordance with current literature [26]. For the ‘Possible’ category, we included patients who presented with typical clinical symptoms and positive Borrelia-specific antibodies in the serum, after the exclusion of other causes, but with no CSF findings. Patients were assigned to the ‘Probable’ category when all criteria for the ‘Possible’ category were met, with the additional evidence of inflammatory CSF syndrome. Lastly, the ‘Definite’ category required meeting all ‘Probable’ criteria, along with confirmation through the intrathecal synthesis of Borrelia-specific antibodies or the positive detection of Borrelia in the CSF by culture or PCR [26].

### 2.3. Diagnostic Procedures

Routine serological workup consisted of a two-step process unless otherwise specified. An initial screening (CLIA using recombinant VIsE antigen, DiaSorin, LIAISON, Italy) was followed by a Western blot (WB) test (Microgen, Neuried, Germany) [27]. For serological diagnosis, EIA/IFA and immunoblot were interpreted according to the NICE guidelines and ESCMID recommendations [28,29]. To assess the potential occurrence of false positive results, antinuclear antibody (ANA) determination was also carried out.

### 2.4. Statistical Analysis

Quantitative variables are reported as mean ± standard deviation (SD), and variables with skewed distributions are presented as median and interquartile range (IQR), as appropriate. All statistical analyses were performed using Stata version 16.1 (StataCorp, College Station, TX, USA).

## 3. Results

### 3.1. Patient Characteristics

We included 130 children. The median age at the time of the first visit was six years (IQR 4–9), and 45% were females. The patient demographics are summarized in Table 1.

Overall, most of the children (80%) had a positive history of tick bites, which occurred between the peak months of April and August in 82% of the cases.

Patients were classified into three definite categories, based upon their symptom’s presentation and referral notes: suspected early localized LB, suspected LB with features other than EM, and asymptomatic tick bite.

One third (32%) of the patients did not show any symptoms but were referred for evaluation after a tick bite. A total of 58 patients (45%) were referred with a suspicion of early localized LB or a history of EM. Half of these presented with atypical EM. 

The remaining 23% presented with features other than EM, the commonest being fever, followed by arthritis and facial nerve palsy. The duration of complaints varied. Other non-specific symptoms included recurrent knee pain, persistent headache, and lymphadenopathy, as reported in Table 2.

### 3.2. Diagnostic Workup and Case Confirmation

Using a composite clinical and serological diagnostic approach, 44/130 (34%) patients were diagnosed with LB, of which 31 (70%) reported a known history of tick bite.

Of the children presenting with EM, 25 (43%) were ultimately diagnosed as having other dermatological lesions; only 33 (57%) were confirmed to have true EM, and, of these, 4 (12%) were atypical. 

A total of 10/33 (30%) patients with confirmed early localized LB had a negative two-tier test, even though the two-tier test was performed prematurely for 3 (30%) of these patients considering the date of the tick bite; 11/33 (33%) patients had a positive two-tier test, while the remaining 12/33 (36%) were not tested. 

All patients with early localized LB received antibiotic treatment: 30 received oral amoxicillin for 14–21 days, 1 patient received azithromycin for 17 days, while 2 patients, aged >8 years, were successfully treated with oral doxycycline for 21 days.

Among the children with confirmed LB, ten (23%) had early disseminated or late disease. Of these, three children with facial nerve palsy were diagnosed as having probable LB based on anamnesis (one had a tick bite, whereas the other two were exposed to ticks during the LB season, and none had a history of herpetic lesions), and all with positive serology. Moreover, one of them had a history of an unrecognized EM which had resolved spontaneously. One child complained of fever, intense headache, confusion, neck pain, and meningism. To confirm the diagnosis of neuroborreliosis, a lumbar puncture was performed, revealing cerebrospinal fluid pleocytosis associated with positive intrathecal *B. burgdorferi* antibody production. Finally, one patient presented with bluish-red swelling on the right ear lobe. A biopsy of the lesion associated with serology ultimately confirmed the clinical suspicion of borrelial lymphocytoma. The detailed clinical findings of confirmed cases with symptoms other than EM are presented in Table 3. 

### 3.3. Serology Findings

Most of the 130 patients who were evaluated (123/130, 95%) received a serological test irrespective of symptoms. Half of them (66/123, 54%) returned a positive screening result. A total of 20 patients were confirmed through positive two-tier testing. Approximately one third of the children with a positive EIA did not receive a confirmatory immunoblot (20/66, 30%); conversely, 20/57 (35%) children with a negative screening test also received a confirmatory immunoblot. No false positive results were found.

In those patients with a known history of tick bite, the interval between the bite and the serology varied, with a median of 32.5 days (IQR 21–52).

### 3.4. Asymptomatic Tick Bite

At the time of our initial evaluation, serological testing had already been conducted in 37 out of the 42 children referred for an asymptomatic tick bite (88%). Only one asymptomatic patient was found positive in the confirmatory immunoblot, but later recalled a skin lesion that was consistent with EM. No asymptomatic infections were documented.

At the time of the first consultation, 18 (43%) patients were already on an empirical antimicrobial regimen with oral amoxicillin in 78% of cases, while the others were on azithromycin, as prescribed by their primary care pediatrician. All inappropriate treatments were stopped.

### 3.5. Spatial–Temporal Distribution of Confirmed Cases

**Figure 1:**
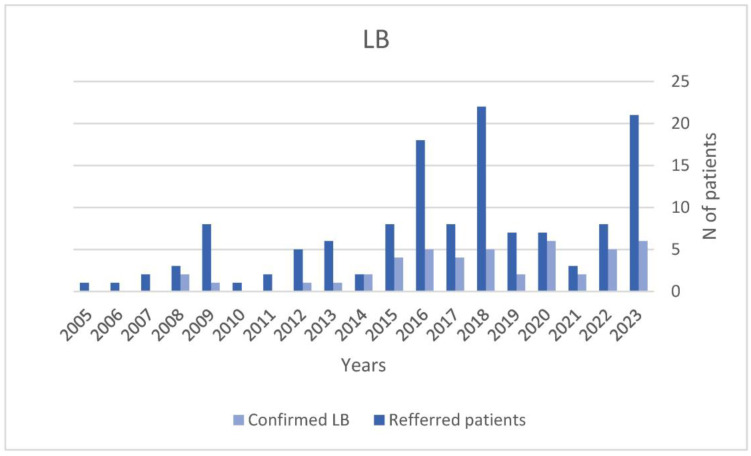
Trend over time for referred and confirmed cases of Lyme Borreliosis (LB).

More than seventy percent of the confirmed LB cases (70%) recalled a history of tick bites and the area where it occurred, which, in 60% of cases, was the peri-urban countryside in Lombardy and around the river Po valley. We also observed an upward trend in referrals over time, except for the years 2020–2021, where the regional healthcare system witnessed a significant decline in specialist consultations due to the impact of the COVID-19 pandemic. For details, see Figure 1.

## 4. Discussion

This study describes a series of 130 cases evaluated for suspected LB at our Pediatric Infectious Disease Unit in Lombardy, a non-endemic region of northern Italy. Of the 130 children who were referred to our center between January 2005 and July 2023, a total of 44 (34%) were ultimately diagnosed with LB.

In our setting, after a tick bite, the primary point of contact is the family pediatrician or the pediatric ED. The main reason for referral was the evaluation of skin lesions deemed suspicious for EM, even in cases without a known history of tick bites or travel to LB endemic areas.

It has been hypothesized that symptoms may vary geographically, and also according to the specific Borrelia genospecies causing the infection, although EM is recognized as the predominant manifestation of LB in childhood [30,31,32,33,34]. Indeed, most LB cases in our study were associated with EM, which is consistent with data from the Italian endemic region of Friuli Venezia Giulia [35]. Based on history and clinical examination, we confirmed a diagnosis of early LB in 57% of the presumptive EM cases.

The timing of the serology showed variability (IQR of 21–52 days from the date of the tick bite, when known). Among the confirmed LB cases, all tests performed earlier than 21 days were negative, confirming the importance of accurate timing for serology to detect seroconversion, and its limited usefulness in the presence of early clinical manifestations of the disease such as EM [20]. Pediatricians working in EDs, who are likely to experience tick removal or the evaluation of skin rashes, should be aware of the limitations of testing too early [36]. In our cohort, WB demonstrated good accuracy; however, in 33% of cases, the recommendations for two-tier testing were not adhered to, and negative serological screening was followed by WB, or WB was not performed after a positive EIA. The need to streamline the diagnostic process, especially at primary healthcare centers that do not have the expertise and laboratory facilities to perform and interpret WB, requires alternatives to two-tier testing, one possibility being a modified two-step algorithm, in which the immunoblot is replaced by a second EIA [22,37,38].

Antibody detection is not recommended in asymptomatic patients, also due to the possibility of false positive results [4,17]. A false positive test, however, should be differentiated from the concept of an asymptomatic patient [39,40]. Asymptomatic seroconversion, that is, the development of a serological response for *B. burgdorferi* without specific signs of the disease, is increasingly being reported; its incidental finding varies from 2.6 to 15% among children in endemic areas [39,41,42]. In our cohort, we found only one positive two-tier testing result in a child referred for an asymptomatic tick bite. In this case, the parents later recalled a past lesion resembling EM, enabling us to confirm a diagnosis of LB.

Little is known about the natural history of LB and the management of asymptomatic infection in children. A careful medical history including any history of tick bite and travel is key to identifying children with a true positive serology who deserve follow-up, given the potential risk of late disease manifestations [39].

In our cohort, almost half of the children evaluated for LB complained of non-specific symptoms, including fatigue, musculoskeletal pain, skin lesions/rash, and persistent headache. The literature is inconsistent regarding the extent to which a borrelial infection can cause persistent, non-specific symptoms [43,44]. Our data showed that non-specific symptoms were observed only in two patients who were ultimately confirmed with LB.

We observed an uptrend in the incidence of LB in our setting. This is consistent with the results of a recent spatiotemporal and risk factor analysis demonstrating that the incidence of LB is increasing in Lombardy, where environmental conditions are suitable for the proliferation of carrier Ixodes ticks [12]. Additionally, another recent study gathered cases of LB from central and southern Italian regions, suggesting that the spread of LB in Italy is higher than previously thought [11]. Whereas Italy has traditionally been regarded as a low-endemicity area for Lyme disease, the lack of mandatory reporting and the absence of recent data from the National Health Institute (Istituto Superiore di Sanità, ISS) are likely contributing to under-reporting [45]. Further epidemiological studies are needed to assess local seroprevalence, and to map hotspots with infected ticks, which are an irreplaceable aid to test interpretation [46].

Compared with data from adult studies [47], the rate of false positive results in our cohort was low, which could be explained by selection bias, since this analysis included mostly children with an intermediate pre-test probability of LB. Due to the low diagnostic accuracy observed in the initial clinical assessments for EM, it is advisable to consider a routine consultation with a specialist in atypical cases, to possibly limit overtreatment or misdiagnosis.

To the best of our knowledge, this study is the largest Lyme disease pediatric report in Italy. Nonetheless, certain limitations should be noted. First, we included children who had been initially assessed by emergency room physicians or by their family pediatrician and tested for LB without a specific indication. This inclusion might have affected the number of negative serological results in this group, as these children had a low pre-test probability of having LB. Second, not all clinical records reported the location of the tick bite, which could have contributed valuable information for geospatial analyses. Last, we did not include an analysis of co-infections with other tick-borne pathogens due to the cohort’s retrospective nature and the timeframe of data collection, spanning 18 years.

## 5. Conclusions

Of the children referred for specialist assessment, only one third were confirmed to have LB; however, the data suggest an increase in diagnoses over time. The strengthening of LB surveillance and notification at the national and European levels is necessary, as our findings suggest that the incidence of LB in children in our region might be higher than previously thought. Increased disease awareness and appropriate diagnostic management are essential. Two-tier testing specificity was high in our setting, but we found frequent deviations from the international guidelines regarding the timing of serology, application of the two-step algorithm, and antibiotic over-prescription. Because most children are primarily assessed for a tick bite or a skin lesion suggestive of EM by a family pediatrician, these findings suggest the importance of implementing training and educational initiatives at the primary healthcare level.

## Figures and Tables

**Table 1:** Patient characteristics.

Total Patients		130
Median age at the time of the visit, years (IQR)		6 (4–9)
Sex	Male	72 (55%)
	Female	58 (45%)
Reason for referral	Suspected early localized Lyme borreliosis Typical EM Atypical EM	58 (45%) 29 (50%) 29 (50%)
	Suspected LD with features other than EM	30 (23%)
	Asymptomatic tick bite	42 (32%)
History of tick bite	Positive April–August	104 (80%) 85 (82%)

Abbreviations: LB, Lyme borreliosis; EM, erythema migrans; IQR, interquartile range.

**Table 2:** Presenting symptoms other than erythema migrans in 30 patients.

Total Patients		30
Fever	Fever	16 (53%)
	Relapsing fever	2 (7%)
Facial nerve palsy		3 (10%)
Arthritis		4 (13%)
Other symptoms	Recurrent joint/musculoskeletal pain	6 (20%)
	Hives	3 (10%)
	Uveitis	1 (3%)
	Lymphadenopathy	3 (10%)
	Abscess	1 (3%)
	Persistent headache	5 (17%)
	Borrelial Lymphocytoma Cutis	1 (3%)

**Table 3:** Clinical characteristics of confirmed cases with symptoms other than erythema migrans.

	Age (in Years), Sex	History of Tick Bite	Presenting Symptoms	History of EM	TTT	Treatment
Patient 1	5, male	Positive	Facial nerve palsy	Negative	Positive	Oral amoxicillin for 21 days
Patient 2	5, male	Negative	Facial nerve palsy	Positive	Positive	Oral amoxicillin for 21 days
Patient 3	3, male	Negative	Facial nerve palsy	Negative	Positive	Oral amoxicillin for 21 days
Patient 4	8, female	Negative	Arthritis	Negative	Positive	Oral doxycycline for 28 days
Patient 5	3, female	Negative	Arthritis	Negative	Positive	Oral amoxicillin for 28 days
Patient 6	12, male	Negative	Arthritis	Negative	Positive	Oral amoxicillin for 28 days
Patient 7	4, male	Positive	Relapsing fever and recurrent knee pain	Negative	Positive	Oral amoxicillin for 28 days
Patient 8	6, male	Positive	Relapsing fever/persistent headache	Negative	Positive	Oral amoxicillin for 28 days
Patient 9	4, male	Negative	Cutaneous B cell pseudolymphomas	Negative	Positive	Oral amoxicillin for 28 days
Patient 10	4, female	Positive	Headache, fever, neck stiffness	Positive	Positive	Ceftriaxone iv for 14 days

Abbreviations: EM, erythema migrans; TTT, two-tier testing: iv, intravenous.

## Data Availability

Most of the important elaborated data generated and analyzed during this study are included in this published article. Raw data will be available on request (by contacting vania.giacomet@unimi.it).
